# On Lightlike Geometry of Para-Sasakian Manifolds

**DOI:** 10.1155/2014/696231

**Published:** 2014-04-28

**Authors:** Bilal Eftal Acet, Selcen Yüksel Perktaş, Erol Kılıç

**Affiliations:** ^1^Faculty of Arts and Sciences, Department of Mathematics, Adıyaman University, 02040 Adıyaman, Turkey; ^2^Faculty of Arts and Sciences, Department of Mathematics, İnönü University, 44280 Malatya, Turkey

## Abstract

We study lightlike hypersurfaces of para-Sasakian manifolds tangent to the characteristic vector field. In particular, we define invariant lightlike hypersurfaces and screen semi-invariant lightlike hypersurfaces, respectively, and give examples. Integrability conditions for the distributions on a screen semi-invariant lightlike hypersurface of para-Sasakian manifolds are investigated. We obtain a para-Sasakian structure on the leaves of an integrable distribution of a screen semi-invariant lightlike hypersurface.

## 1. Introduction

It is well known that the main difference between the geometry of submanifolds in Riemannian manifolds and in semi-Riemannian manifolds is that in the latter case the induced metric tensor field by the semi-Riemannian metric on the ambient space is not necessarily nondegenerate. If the induced metric tensor field is degenerate the classical theory of Riemannian submanifolds fails since the normal bundle and the tangent bundle of the submanifold have a nonzero intersection. In particular, from the point of physics lightlike hypersurfaces are important as they are models of various types of horizons, such as Killing, dynamical and conformal horizons, studied in general relativity.

Lightlike submanifolds of semi-Riemannian manifolds were introduced by Duggal and Bejancu in [1]. Since then many authors studied lightlike hypersurfaces of semi-Riemannian manifolds and especially of indefinite Sasakian manifolds (for differential geometry of lightlike submanifolds we refer to the book [2]).

The study of paracontact geometry was initiated by Kaneyuki and Konzai in [3]. The authors defined almost paracontact structure on a pseudo-Riemannian manifold *M* of dimension (2*n* + 1) and constructed the almost paracomplex structure on *M* ^2*n*+1^ × *R*. Recently, Zamkovoy [4] studied paracontact metric manifolds and some remarkable subclasses like para-Sasakian manifolds. In particular, in the recent years, many authors [5–9] have pointed out the importance of paracontact geometry and, in particular, of para-Sasakian geometry, by several papers giving the relationships with the theory of para-Kähler manifolds and its role in pseudo-Riemannian geometry and mathematical physics.

These circumstances motivated us to initiate the study of lightlike geometry of submanifolds in almost paracontact metric manifolds. As a first step, in the present paper, we study the lightlike hypersurfaces of almost paracontact metric manifolds. We introduce the invariant lightlike hypersurfaces and screen semi-invariant lightlike hypersurfaces of almost paracontact metric manifolds, respectively, and give examples. Moreover, integrability conditions for the distributions involved in the definition of a screen semi-invariant lightlike hypersurface are investigated in case of the ambient manifold being para-Sasakian manifold.

## 2. Preliminaries

### 2.1. Almost Paracontact Metric Manifolds

A differentiable manifold $\overset{-}{M}$ of dimension (2*n* + 1) is called almost paracontact manifold with the almost paracontact structure $\left(\overset{-}{\phi },\xi ,\eta \right)$ if it admits a tensor field $\overset{-}{\phi }$ of type (1,1), a vector field *ξ*, and a 1-form *η* satisfying the following conditions [3]:

(1) $$\begin{array}{c} {\overset{-}{\phi }}^{2}=I-\eta ⊗\xi , \end{array}$$

(2) $$\begin{array}{c} \eta \left(\xi \right)=1, \end{array}$$

(3) $$\begin{array}{c} \overset{-}{\phi }\xi =0, \end{array}$$

(4) $$\begin{array}{c} \eta \circ \overset{-}{\phi }=0, \end{array}$$

where *I* denotes the identity transformation. Moreover, the tensor field $\overset{-}{\phi }$ induces an almost paracomplex structure on the paracontact distribution *D* = *ker*⁡*η*; that is, the eigen distributions *D* ^±^ corresponding to the eigenvalues ±1 of $\overset{-}{\phi }$ are both *n*-dimensional.

If a (2*n* + 1)-dimensional almost paracontact manifold $\overset{-}{M}$ with an almost paracontact structure $\left(\overset{-}{\phi },\xi ,\eta \right)$ admits a pseudo-Riemannian metric $\overset{-}{g}$ such that [4]

(5) $$\begin{array}{c} \overset{-}{g}\left(\overset{-}{\phi }X,\overset{-}{\phi }Y\right)=-\overset{-}{g}\left(X,Y\right)+\eta \left(X\right)\eta \left(Y\right),\;X,Y\in \Gamma \left(T\overset{-}{M}\right), \end{array}$$

then we say that $\overset{-}{M}$ is an* almost paracontact metric manifold* with an* almost paracontact metric structure* $\left(\overset{-}{\phi },\xi ,\eta ,\overset{-}{g}\right)$ and such metric $\overset{-}{g}$ is called* compatible metric*. Any compatible metric $\overset{-}{g}$ is necessarily of signature (*n* + 1, *n*).

From (5) it can be easily seen that [4]

(6) $$\begin{array}{c} \overset{-}{g}\left(\overset{-}{\phi }X,Y\right)=-\overset{-}{g}\left(X,\overset{-}{\phi }Y\right), \end{array}$$

(7) $$\begin{array}{c} \overset{-}{g}\left(X,\xi \right)=\eta \left(X\right), \end{array}$$

for any $X,Y\in \Gamma (T\overset{-}{M})$. The fundamental 2-form of $\overset{-}{M}$ is defined by

(8) $$\begin{array}{c} \Phi \left(X,Y\right)=\overset{-}{g}\left(X,\overset{-}{\phi }Y\right). \end{array}$$

An almost paracontact metric structure becomes a paracontact metric structure if $\overset{-}{g}(X,\overset{-}{\phi }Y)=d\eta (X,Y)$, for all vector fields $X,Y\in \Gamma (T\overset{-}{M})$, where *dη*(*X*, *Y*) = (1/2){*Xη*(*Y*) − *Yη*(*X*) − *η*([*X*, *Y*])}.

For a (2*n* + 1)-dimensional manifold $\overset{-}{M}$ with an almost paracontact metric structure $\left(\overset{-}{\phi },\xi ,\eta ,\overset{-}{g}\right)$ one can also construct a local orthonormal basis which is called $\overset{-}{\phi }$-*basis* $\left(X_{i},\overset{-}{\phi }X_{i},\xi )\;\;(i=1,2,\ldots ,n\right)$ [4].

An almost paracontact metric structure $\left(\overset{-}{\phi },\xi ,\eta ,\overset{-}{g}\right)$ is a* para-Sasakian manifold* if and only if [4]

(9) $$\begin{array}{c} \left({\overset{-}{\nabla }}_{X}\overset{-}{\phi }\right)Y=-\overset{-}{g}\left(X,Y\right)\xi +\eta \left(Y\right)X,\;X,Y\in \Gamma \left(T\overset{-}{M}\right), \end{array}$$

where $X,Y\in \Gamma (T\overset{-}{M})$ and $\overset{-}{\nabla }$ is a Levi-Civita connection on $\overset{-}{M}$.

From (9), it can be seen that

(10) $$\begin{array}{c} {\overset{-}{\nabla }}_{X}\xi =-\overset{-}{\phi }X. \end{array}$$

**Example 1**

Let $\overset{-}{M}=R^{2n+1}$ be the (2*n* + 1)-dimensional real number space with standard coordinate system (*x*_1_, *y*_1_, *x*_2_, *y*_2_,…, *x*_*n*_, *y*_*n*_, *z*). Defining

(11) $$\begin{array}{c} \bar{\phi }\frac{\partial }{\partial x_{\alpha }}=\frac{\partial }{\partial y_{\alpha }},\;\;\bar{\phi }\frac{\partial }{\partial y_{\alpha }}=\frac{\partial }{\partial x_{\alpha }},\;\;\bar{\phi }\frac{\partial }{\partial z}=0,\\ \xi =\frac{\partial }{\partial z},\;\;\bar{\eta }=dz,\\ \bar{g}=\eta ⊗\eta +\overset{n}{\underset{\alpha =1}{\sum }}dx_{\alpha }⊗dx_{\alpha }-\overset{n}{\underset{\alpha =1}{\sum }}dy_{\alpha }⊗dy_{\alpha }, \end{array}$$

where *α* = 1,2,…, *n*, the set $\left(\overset{-}{\phi },\xi ,\eta ,\overset{-}{g}\right)$ is an almost paracontact metric structure on *R* ^2*n*+1^.

### 2.2. Lightlike Hypersurfaces

In this section, we recall some basic results about lightlike hypersurfaces of a semi-Riemannian manifold $\left(\overset{-}{M},\overset{-}{g}\right)$ [1].

Let $\left(\overset{-}{M},\overset{-}{g}\right)$ be a (*n* + 1)-dimensional semi-Riemannian manifold with index *q*  (0 < *q* < *n* + 1) and *M* a hypersurface of $\overset{-}{M}$. Assume that the induced metric $g=\overset{-}{g}{|}_{M}$ on the hypersurface is degenerate on *M*. Then there exists a vector field *E* ≠ 0 on *M* such that

(12) $$\begin{array}{c} g\left(E,X\right)=0,\;\forall X\in \Gamma \left(TM\right). \end{array}$$

The radical space [10] of *T*_*p*_ *M* is defined by

(13) $$\begin{array}{c} \text{Rad}\;T_{p}M=\left\{E\in \text{Rad}\;T_{p}M:g\left(E,X\right)=0,\forall X\in \Gamma \left(T_{p}M\right)\right\}, \end{array}$$

whose dimension is called the nullity degree of *g* and (*M*, *g*) is called* a lightlike hypersurface* of $\left(\overset{-}{M},\overset{-}{g}\right)$. Since *g* is degenerate and any null vector is perpendicular to itself, *T*_*p*_ *M* ^⊥^ is also degenerate and

(14) $$\begin{array}{c} \text{Rad}\;T_{p}M=T_{p}M\cap T_{p}M^{⊥}. \end{array}$$

For a lightlike hypersurface *M*, dim⁡*T*_*p*_ *M* ^⊥^ = 1 implies that

(15) $$\begin{array}{c} \dim\left(\text{Rad}\;T_{p}M\right)=1,\;\;\text{Rad}\;T_{p}M=T_{p}M^{⊥}. \end{array}$$

We call Rad *T*_*p*_ *M* the radical distribution and it is spanned by the null vector field *E*.

Consider complementary vector bundle *S*(*TM*) of Rad *TM* in *TM*. This means that

(16) $$\begin{array}{c} TM=S\left(TM\right)⊥\text{Rad}\;TM, \end{array}$$

where ⊥ denotes the orthogonal direct sum. *S*(*TM*) is called the* screen distribution* on *M*. Since the screen distribution *S*(*TM*) is nondegenerate, there exists a complementary orthogonal vector subbundle *S*(*TM*)^⊥^ to *S*(*TM*) in $T\overset{-}{M}$ which is called* screen transversal subbundle*; that is,

(17) $$\begin{array}{c} T\overset{-}{M}=S\left(TM\right)⊥S{\left(TM\right)}^{⊥}. \end{array}$$

The rank of *S*(*TM*)^⊥^ is 2.

**Theorem 2 (see [1])**

Let (*M*, *g*, *S*(*TM*)) be a lightlike hypersurface of an almost paracontact manifold $\left(\overset{-}{M},\overset{-}{\phi },\xi ,\eta ,\overset{-}{g}\right)$. Then there exists a unique rank one vector subbundle ltr(*TM*) of  $T\overset{-}{M}$, with base space *M*, such that, for any nonzero section *E* of Rad *TM* on a coordinate neighborhood *U* ⊂ *M*, there exists a unique section *N* of ltr(*TM*) on *U* satisfying

(18) $$\begin{array}{c} \overset{-}{g}\left(N,E\right)=1,\;\;\overset{-}{g}\left(N,N\right)=0,\;\;\overset{-}{g}\left(N,W\right)=0, \end{array}$$

for all *W* ∈ Γ(*S*(*TM*))|_*U*_. ltr(*TM*) is called the lightlike transversal vector bundle of *M* with respect to *S*(*TM*).

One can consider the following decompositions:

(19) $$\begin{array}{c} S{\left(TM\right)}^{⊥}=\text{Rad}\;TM⊕\text{ltr}\left(TM\right), \end{array}$$

(20) TM−|M=S(TM)⊥{Rad  TM⊕ltr(TM)}=TM⊕ltr(TM).

Let $\overset{-}{\nabla }$ be the Levi-Civita connection on $\overset{-}{M}$. Using (20) we deduce

(21) $$\begin{array}{c} {\overset{-}{\nabla }}_{X}Y=\nabla_{X}Y+h\left(X,Y\right), \end{array}$$

(22) $$\begin{array}{c} {\overset{-}{\nabla }}_{X}N=-A_{N}X+\nabla_{X}^{t}N, \end{array}$$

for any *X*, *Y* ∈ Γ(*TM*) and *N* ∈ Γ(ltr(*TM*)). Then $\overset{-}{\nabla }$ and ∇^*t*^ are called the induced connection on *M* and ltr(*TM*), respectively, and, as in the classical theory of Riemannian hypersurfaces, *h* and *A*_*N*_ are called the second fundamental form and the shape operator, respectively. The above equations are cited as the Gauss and Weingarten equation, respectively [1].

Locally, let *E*, *N*, and *U* be as in Theorem 2. Then, for any *X*, *Y* ∈ Γ(*TM*|_*U*_), putting

(23) $$\begin{array}{c} B\left(X,Y\right)=\overset{-}{g}\left(h\left(X,Y\right),E\right),\;\;\tau \left(X\right)=\overset{-}{g}\left(\nabla_{X}^{t}N,E\right), \end{array}$$

we can write

(24) $$\begin{array}{c} {\overset{-}{\nabla }}_{X}Y=\nabla_{X}Y+B\left(X,Y\right)N, \end{array}$$

(25) $$\begin{array}{c} {\overset{-}{\nabla }}_{X}N=-A_{N}X+\tau \left(X\right)N. \end{array}$$

*B* is called the local second fundamental form of *M*, because it determines *h* on *U*. Moreover, *B* is degenerate and

(26) $$\begin{array}{c} B\left(X,E\right)=0, \end{array}$$

for any *X* ∈ Γ(*TM*|_*U*_).

The decomposition (16) allows to define a canonical projection *P* : Γ(*TM*) → Γ(*S*(*TM*)). For each *X* ∈ Γ(*TM*), we may write

(27) $$\begin{array}{c} X=PX+\theta \left(X\right)E, \end{array}$$

where *θ* is a 1-form given by

(28) $$\begin{array}{c} \theta \left(X\right)=\overset{-}{g}\left(X,N\right). \end{array}$$

From (24), for all *X*, *Y*, *Z* ∈ Γ(*TM*), we get

(29) $$\begin{array}{c} \left(\nabla_{X}g\right)\left(Y,Z\right)=B\left(X,Y\right)\theta \left(Z\right)+B\left(X,Z\right)\theta \left(Y\right), \end{array}$$

which implies that the induced connection ∇ is a nonmetric connection on *M*.

Then for any *X*, *Y* ∈ Γ(*TM*) and *E* ∈ Γ(*TM* ^⊥^) we can write

(30) $$\begin{array}{c} \nabla_{X}PY=\nabla_{X}^{*}PY+h^{*}\left(X,PY\right), \end{array}$$

(31) $$\begin{array}{c} \nabla_{X}E=-A_{E}^{*}X+\nabla_{X}^{*t}E, \end{array}$$

where ∇* and ∇^∗*t*^ are linear connections on the bundles *S*(*TM*) and Rad(*TM*), respectively. Further, h* and A* are called the second fundamental form and the shape operator of the screen distribution, respectively. Locally, let *U* be a coordinate neighborhood of *M* and *E*, *N* sections on *U*, as in Theorem 2. Then, putting $C(X,PY)=\overset{-}{g}(h^{*}(X,PY),N)$, for any *X*, *Y* ∈ Γ(*TM*|_*U*_), one has

(32) $$\begin{array}{c} h^{*}\left(X,PY\right)=C\left(X,PY\right)E,\;\;\overset{-}{g}\left(\nabla_{X}^{*t}E,N\right)=-\tau \left(X\right), \end{array}$$

and, locally on *U*, (30) and (31) become

(33) $$\begin{array}{c} \nabla_{X}PY=\nabla_{X}^{*}PY+C\left(X,PY\right)E,\\ \nabla_{X}E=-A_{E}^{*}X-\tau \left(X\right)E. \end{array}$$

The local second fundamental forms *B* and *C*, respectively, of *M* and on *S*(*TM*) are related to their shape operators by

(34) $$\begin{array}{c} g\left(A_{E}^{*}X,PY\right)=B\left(X,PY\right),\;\;g\left(A_{E}^{*}X,N\right)=0, \end{array}$$

(35) $$\begin{array}{c} g\left(A_{N}X,PY\right)=C\left(X,PY\right),\;\;g\left(A_{N}X,N\right)=0. \end{array}$$

Furthermore, one has *A*_E_*X = 0, ${\overset{-}{\nabla }}_{E}E=\nabla_{E}E=-\tau (E)E$. (For more details we refer to [1, 2].)

## 3. Lightlike Hypersurfaces of Para-Sasakian Manifolds

Let $\left(\overset{-}{M},\overset{-}{\phi },\xi ,\eta ,\overset{-}{g}\right)$ be a (2*n* + 1)-dimensional para-Sasakian manifold and *M* a lightlike hypersurface of $\overset{-}{M}$ such that the structure vector field *ξ* is tangent to *M*. For local sections *E* and *N* of Rad(*TM*) and ltr(*TM*), respectively, in view of (7) we have

(36) $$\begin{array}{c} \eta \left(E\right)=0,\;\;\eta \left(N\right)=0. \end{array}$$

From (6) it is easy to see that $\overset{-}{\phi }E$ and $\overset{-}{\phi }N$ are lightlike vector fields and

(37) $$\begin{array}{c} {\overset{-}{\phi }}^{2}E=E,\;\;{\overset{-}{\phi }}^{2}N=N. \end{array}$$

Now, for any *X* ∈ Γ(*TM*), we write

(38) $$\begin{array}{c} \overset{-}{\phi }X=\phi X+u\left(X\right)N, \end{array}$$

where *ϕX* ∈ Γ(*TM*) and

(39) $$\begin{array}{c} u\left(X\right)=\overset{-}{g}\left(\overset{-}{\phi }X,E\right)=-\overset{-}{g}\left(X,\overset{-}{\phi }E\right). \end{array}$$

**Proposition 3**

Let $\left(\overset{-}{M},\overset{-}{\phi },\xi ,\eta ,\overset{-}{g}\right)$ be a (2*n* + 1)-dimensional para-Sasakian manifold and *M* a lightlike hypersurface of $\overset{-}{M}$ such that the structure vector field *ξ* is tangent to *M*. Then one has

(40) $$\begin{array}{c} \overset{-}{g}\left(\overset{-}{\phi }E,N\right)=-g\left(A_{N}E,\xi \right), \end{array}$$

where *E* is any local section of Rad(*TM*) and *N* is any local section of ltr(*TM*).

**Proof**

From (10) and (18) we have

(41) $$\begin{array}{c} \overset{-}{g}\left(\overset{-}{\phi }E,N\right)=-\overset{-}{g}\left({\overset{-}{\nabla }}_{E}\xi ,N\right)=\overset{-}{g}\left(\xi ,{\overset{-}{\nabla }}_{E}N\right), \end{array}$$

which gives (40) by virtue of (22).

**Remark 4**

From (6) we get $\overset{-}{g}\left(\overset{-}{\phi }E,E\right)=0$, which implies that there is no component of $\overset{-}{\phi }E$ in ltr(*TM*) and so $\overset{-}{\phi }E\in \Gamma (TM)$. Moreover, (40) implies that there may be a component of $\overset{-}{\phi }E$ in Rad(*TM*). Thus, in view of (27), we observe that

(42) $$\begin{array}{c} \overset{-}{\phi }E=\phi E=P\overset{-}{\phi }E+\theta \left(\overset{-}{\phi }E\right)E. \end{array}$$

**Proposition 5**

Let $\left(\overset{-}{M},\overset{-}{\phi },\xi ,\eta ,\overset{-}{g}\right)$ be a (2*n* + 1)-dimensional para-Sasakian manifold and *M* a lightlike hypersurface of $\overset{-}{M}$ such that the structure vector field *ξ* is tangent to *M*. Then one has

(43) $$\begin{array}{c} g\left(X,\phi Y\right)=-g\left(\phi X,Y\right)-\left(u⊗\theta +\theta ⊗u\right)\left(X,Y\right), \end{array}$$

(44) g(ϕX,ϕY)=−g(X,Y)+η(X)η(Y)−u(X)θ(ϕY)−u(Y)θ(ϕX),

for any *X*, *Y* ∈ Γ(*TM*).

**Proof**

By using (38) and (39) we obtain

(45) $$\begin{array}{c} \overset{-}{g}\left(\overset{-}{\phi }X,Y\right)=g\left(X,\phi Y\right)+u\left(Y\right)g\left(X,N\right). \end{array}$$

Hence in view of (6) we get (43). From (38) we have

(46) $$\begin{array}{c} \overset{-}{g}\left(\overset{-}{\phi }X,\overset{-}{\phi }Y\right)=g\left(\phi X,\phi Y\right)+u\left(Y\right)\theta \left(\phi X\right)+u\left(X\right)\theta \left(\phi Y\right). \end{array}$$

Thus, by using the last equation above and (5), we complete the proof.

**Corollary 6**

Let *M* be a lightlike hypersurface of a para-Sasakian manifold such that the structure vector field *ξ* is tangent to *M*. Then, for all *X* ∈ Γ(*TM*), one has *g*(*ξ*, *ϕX*) = 0.

**Proposition 7**

Let *M* be a lightlike hypersurface of a (2*n* + 1)-dimensional para-Sasakian manifold $\left(\overset{-}{M},\overset{-}{\phi },\xi ,\eta ,\overset{-}{g}\right)$ such that the structure vector field *ξ* is tangent to *M*. Then, for any *X* ∈ Γ(*TM*), one has

(47) $$\begin{array}{c} \phi^{2}X=X-\eta \left(X\right)\xi -u\left(\phi X\right)N-u\left(X\right)\overset{-}{\phi }N, \end{array}$$

(48) $$\begin{array}{c} \nabla_{X}\xi =-\phi X, \end{array}$$

(49) $$\begin{array}{c} B\left(X,\xi \right)=-u\left(X\right). \end{array}$$

**Proof**

From (1) and (38), we get (47). Next, by using (10), (24), and (38), we have

(50) $$\begin{array}{c} \nabla_{X}\xi +B\left(X,\xi \right)=-\phi X-u\left(X\right)N. \end{array}$$

Then by equating the tangential and transversal parts in the previous equation we get (48) and (49), respectively.

## 4. Invariant Lightlike Hypersurfaces of Almost Paracontact Metric Manifolds

We begin with the following.

**Definition 8**

Let $\left(\overset{-}{M},\overset{-}{\phi },\xi ,\eta ,\overset{-}{g}\right)$ be a (2*n* + 1)-dimensional para-Sasakian manifold and *M* a lightlike hypersurface of $\overset{-}{M}$ such that the structure vector field *ξ* is tangent to *M*. If $\overset{-}{\phi }\left(S(TM)\right)\subseteq S(TM)$, then *M* will be called an invariant lightlike hypersurface of $\overset{-}{M}$.

**Example 9**

Let $\overset{-}{M}=R^{5}$ be the 5-dimensional real number space with a coordinate system (*x*_1_, *y*_1_, *x*_2_, *y*_2_, *z*). Define a frame of vector fields on $\overset{-}{M}$ given by We*ƚ*yczko [11]:

(51) $$\begin{array}{c} e_{1}=\frac{\partial }{\partial x_{1}},\;\;e_{2}=\frac{\partial }{\partial x_{2}},\\ e_{3}=\frac{1}{1+y_{1}^{2}}\frac{\partial }{\partial x_{1}}+\frac{\partial }{\partial y_{1}}-2x_{1}\frac{\partial }{\partial z},\\ e_{4}=\frac{1}{1+y_{1}^{2}}\frac{\partial }{\partial x_{2}}+\frac{\partial }{\partial y_{2}}-2x_{2}\frac{\partial }{\partial z},\\ e_{5}=\frac{\partial }{\partial z}. \end{array}$$

Defining

(52) η=2x1dy1+2x2dy2+dz,  ξ=e5,ϕ−e1=−e1,  ϕ−e2=−e2,  ϕ−e3=e3,ϕ−e4=e4,  ϕ−e5=e5,g¯(e1,e3)=g¯(e3,e1)=g¯(e2,e4)=g¯(e4,e2)=g¯(e5,e5)=1,g¯(ei,ej)=0, otherwise,

the set $\left(\overset{-}{\phi },\xi ,\eta ,\overset{-}{g}\right)$ is an almost paracontact metric structure on $\overset{-}{M}$ with index $\left(\overset{-}{g}\right)=2$. Consider a hypersurface *M* of $\overset{-}{M}$ given by

(53) $$\begin{array}{c} x_{1}=\arctan y_{1}. \end{array}$$

It is easy to check that *M* is a lightlike hypersurface and

(54) $$\begin{array}{c} \text{Rad}\left(TM\right)=\mathrm{Span}\left\{E=e_{3}\right\}. \end{array}$$

Then the lightlike transversal vector bundle ltr(*TM*) is spanned by

(55) $$\begin{array}{c} N=e_{1}. \end{array}$$

It follows that corresponding screen distribution *S*(*TM*) is spanned by

(56) $$\begin{array}{c} \left\{W_{1}=e_{2},W_{2}=e_{4},W_{3}=e_{5}\right\}. \end{array}$$

We easily check that

(57) $$\begin{array}{c} \overset{-}{\phi }W_{1}=-W_{1},\;\;\overset{-}{\phi }W_{2}=W_{2},\;\;\overset{-}{\phi }W_{3}=0, \end{array}$$

which gives $\overset{-}{\phi }\left(S(TM)\right)\subseteq S(TM)$. Thus *M* is an invariant lightlike hypersurface of $\overset{-}{M}$.

Now we give a characterization of an invariant lightlike hypersurface.

**Theorem 10**

Let $\left(\overset{-}{M},\overset{-}{\phi },\xi ,\eta ,\overset{-}{g}\right)$ be a (2*n* + 1)-dimensional para-Sasakian manifold and *M* a lightlike hypersurface of $\overset{-}{M}$ such that the structure vector field *ξ* is tangent to *M*. Then *M* is an invariant lightlike hypersurface of $\overset{-}{M}$ if and only if

(58) $$\begin{array}{c} \overset{-}{\phi }\text{Rad}\;TM=\text{Rad}\;TM,\;\;\overset{-}{\phi }\text{ltr}TM=\text{ltr}TM. \end{array}$$

**Proof**

Let *M* be an invariant lightlike hypersurface of $\overset{-}{M}$. From (27) and (42), for any *X* ∈ Γ(*TM*), we get $g(P\overset{-}{\phi }E,PX)=0$; that is, there is no component of $\overset{-}{\phi }E$ in *S*(*TM*). Moreover, it is obvious from (6) that $\overset{-}{\phi }E$ has no component in ltr *TM* and so $\overset{-}{\phi }\text{Rad}\;TM=\text{Rad}\;TM$.

On the other hand, for any local section *N* of ltr *TM*, we can write

(59) $$\begin{array}{c} \overset{-}{\phi }N=P\overset{-}{\phi }N+\overset{-}{g}\left(\overset{-}{\phi }N,E\right)N. \end{array}$$

By using the previous equation we have $g(P\overset{-}{\phi }N,PX)=0$, for any *X* ∈ Γ(*TM*), which implies that $\overset{-}{\phi }N$ has no component in *S*(*TM*). Since $\bar{g}(\overset{-}{\phi }N,N)=0$, then it is also seen that there is no component of $\overset{-}{\phi }N$ in Rad *TM*. Hence $\overset{-}{\phi }\text{ltr}\;TM=\text{ltr}\;TM$.

Conversely, let $\overset{-}{\phi }\text{Rad}\;TM=\text{Rad}TM$ and $\overset{-}{\phi }\text{ltr}TM=\text{ltr}TM$. For any *X* ∈ Γ(*S*(*TM*)) we have

(60) $$\begin{array}{c} \bar{g}\left(\overset{-}{\phi }X,E\right)=-\bar{g}\left(X,\overset{-}{\phi }E\right)=0, \end{array}$$

which implies that $\overset{-}{\phi }X$ has no component in ltr *TM*. Similarly, we get

(61) $$\begin{array}{c} \bar{g}\left(\overset{-}{\phi }X,N\right)=-\bar{g}\left(X,\overset{-}{\phi }N\right)=0; \end{array}$$

thus there is no component of $\overset{-}{\phi }X$ in Rad *TM*. The proof is completed.

**Corollary 11**

Let $\left(\overset{-}{M},\overset{-}{\phi },\xi ,\eta ,\overset{-}{g}\right)$ be a (2*n* + 1)-dimensional para-Sasakian manifold and *M* a lightlike hypersurface of $\overset{-}{M}$ such that the structure vector field *ξ* is tangent to *M*. Then *M* is an invariant lightlike hypersurface of $\overset{-}{M}$ if and only if

(62) $$\begin{array}{c} \overset{-}{\phi }E=\pm E,\;\;\overset{-}{\phi }N=\pm N. \end{array}$$

**Theorem 12**

Let $\left(\overset{-}{M},\overset{-}{\phi },\xi ,\eta ,\overset{-}{g}\right)$ be a (2*n* + 1)-dimensional almost paracontact metric manifold and *M* an invariant lightlike hypersurface of $\overset{-}{M}$. Then (*M*, *ϕ*, *ξ*, *η*, *g*) is an almost paracontact metric manifold.

**Proof**

Let *M* be an invariant lightlike hypersurface of $\bar{M}$. For any *X*, *Y* ∈ Γ(*TM*), from (38), we get

(63) $$\begin{array}{c} \overset{-}{\phi }X=\phi X. \end{array}$$

Using (1) and (63), we have

(64) $$\begin{array}{c} \phi^{2}X=X-\eta \left(X\right)\xi . \end{array}$$

Also from (63), it follows that

(65) $$\begin{array}{c} \phi \xi =0. \end{array}$$

Next, in view of (64) and (65) one can easily see that

(66) $$\begin{array}{c} \eta \circ \phi =0,\\ \eta \left(\xi \right)=1. \end{array}$$

Moreover, from (44), we have

(67) $$\begin{array}{c} g\left(\phi X,\phi Y\right)=-g\left(X,Y\right)+\eta \left(X\right)\eta \left(Y\right). \end{array}$$

From (64)-(67) we complete the proof.

**Proposition 13**

Let *M* be an invariant lightlike hypersurface of a para-Sasakian manifold $\left(\overset{-}{M},\overset{-}{\phi },\xi ,\eta ,\overset{-}{g}\right)$. Then we have

(68) $$\begin{array}{c} g\left(A_{N}X,\xi \right)=0, \end{array}$$

for any *X* ∈ Γ(*TM*).

**Proof**

Since *g*(*ξ*, *N*) = 0, using (10), we get

(69) $$\begin{array}{c} \bar{g}\left({\bar{\nabla }}_{X}N,\xi \right)=\bar{g}\left(N,\bar{\phi }X\right). \end{array}$$

From (25), we have the assertion of the proposition.

**Theorem 14**

An invariant lightlike hypersurface of a para-Sasakian manifold is always para-Sasakian. Moreover,

(70) $$\begin{array}{c} B\left(X,\phi Y\right)N-B\left(X,Y\right)\phi N=0, \end{array}$$

(71) $$\begin{array}{c} \phi \left(A_{N}X\right)=A_{\phi N}X-\theta \left(X\right)\xi , \end{array}$$

for any *X*, *Y* ∈ Γ(*TM*).

**Proof**

We have

(72) (∇¯Xϕ¯)Y=∇¯Xϕ¯Y−ϕ¯(∇¯XY)=∇¯XϕY−ϕ¯(∇XY+B(X,Y)N)=∇XϕY+B(X,ϕY)N−ϕ∇XY−B(X,Y)ϕN=(∇Xϕ)Y+B(X,ϕY)N−B(X,Y)ϕN,

which in view of (9) gives

(73) −g(X,Y)ξ+η(Y)X=(∇Xϕ)Y+B(X,ϕY)N−B(X,Y)ϕN.

Equating tangential parts in (73) provides

(74) $$\begin{array}{c} \left(\nabla_{X}\phi \right)Y=-\;g\left(X,Y\right)\xi +\eta \left(Y\right)X. \end{array}$$

In view of (74) and Theorem 12, we see that *M* is a para-Sasakian manifold. Equating transversal parts in (73) yields (70). Next, using (9) and (25), we have

(75) −θ(X)ξ=(∇¯Xϕ¯)N=∇¯Xϕ¯N−ϕ¯(∇¯XN)=−Aϕ¯NX+g¯(∇¯Xϕ¯N,E)N+ϕ¯(ANX)−τ(X)ϕ¯N.

In the last equation, if we equate the tangential parts, we get (71).

This completes the proof.

**Remark 15**

It is well known that, if there exists a lightlike hypersurface in an indefinite Sasakian manifold, then the dimension of the indefinite Sasakian manifold must be equal or greater than 5. But in a paracontact metric manifold there is not such a restriction in the dimension of the ambient manifold for the existence of lightlike hypersurfaces.

Let $\left(\overset{-}{M},\overset{-}{\phi },\xi ,\eta ,\overset{-}{g}\right)$ be a 3-dimensional almost paracontact metric manifold and *M* a lightlike surface of $\overset{-}{M}$ such that the structure vector field *ξ* is tangent to *M*. Since *ξ* is a nonnull vector field, it belongs to the screen distribution *S*(*TM*). Thus, {*ξ*, *E*, *N*} is a quasi-orthonormal frame of $T\overset{-}{M}$. Also with respect to the quasi-orthonormal frame {*ξ*, *E*, *N*} of $T\overset{-}{M}$ we can write

(76) $$\begin{array}{c} \overset{-}{\phi }E=a\xi +bE+cN,\\ \overset{-}{\phi }N=d\xi +eE+fN, \end{array}$$

where $a=\bar{g}\left(\overset{-}{\phi }E,\xi \right)=0$, $b=\bar{g}\left(\overset{-}{\phi }E,N\right)$, $c=\bar{g}\left(\overset{-}{\phi }E,E\right)=0$, $d=\bar{g}\left(\overset{-}{\phi }N,\xi \right)=0$, $e=\bar{g}\left(\overset{-}{\phi }N,N\right)=0$, and $f=\bar{g}\left(\overset{-}{\phi }E,N\right)=b$. Thus we have

(77) $$\begin{array}{c} \overset{-}{\phi }E=bE,\\ \overset{-}{\phi }N=bN. \end{array}$$

Hence, a lightlike surface *M* of $\overset{-}{M}$, tangent to the structure vector field *ξ*, is always an invariant lightlike surface.

**Example 16**

Let $\overset{-}{M}=R^{3}$ be a 3-dimensional almost paracontact metric manifold with the structure $\left(\overset{-}{\phi },\xi ,\eta ,\overset{-}{g}\right)$ given in Example 1. Consider a surface *M* of $\overset{-}{M}$ given by *x*_1_ = *y*_1_. It is easy to check that *M* is a lightlike surface and Rad(*TM*) and ltr(*TM*) are given by

(78) $$\begin{array}{c} \text{Rad}\;TM=\mathrm{Span}\left\{E=\frac{\partial }{\partial x_{1}}+\frac{\partial }{\partial y_{1}}\right\},\\ \text{ltr}\;TM=\mathrm{Span}\left\{N=\frac{1}{2}\left(\frac{\partial }{\partial x_{1}}-\frac{\partial }{\partial y_{1}}\right)\right\}, \end{array}$$

respectively. It follows that corresponding screen distribution is spanned by

(79) $$\begin{array}{c} \left\{\xi =\frac{\partial }{\partial z}\right\}. \end{array}$$

Then $\overset{-}{\phi }E=E$ and $\overset{-}{\phi }N=-N$, which imply that *M* is an invariant lightlike surface.

**Example 17**

Let (*J*, *G*) be the standard flat para-Kähler structure on *R*_*n*_^2*n*+2^,

(80) $$\begin{array}{c} J\left(\frac{\partial }{\partial x_{\alpha }}\right)=\frac{\partial }{\partial x_{\alpha +n+1}},\;\;J\left(\frac{\partial }{\partial x_{\alpha +n+1}}\right)=\frac{\partial }{\partial x_{\alpha }},\\ G\left(\frac{\partial }{\partial x_{\alpha }},\frac{\partial }{\partial x_{\alpha }}\right)=1,\;\;G\left(\frac{\partial }{\partial x_{\alpha +n+1}},\frac{\partial }{\partial x_{\alpha +n+1}}\right)=-1, \end{array}$$

for *α* = 1,…, *n*, where (*x*_1_, *x*_2_,…, *x*_2*n*+2_) are the Cartesian coordinates on *R*_*n*_^2*n*+2^. Consider the hypersurface *H*_*n*_^2*n*+1^ in *R*_*n*+1_^2*n*+2^ given by the equation

(81) $$\begin{array}{c} H_{n}^{2n+1}=\left\{x\in R_{n+1}^{2n+2}:\overset{n+1}{\underset{\alpha =1}{\sum }}x_{a}^{2}-\overset{2n+2}{\underset{\alpha =n+2}{\sum }}x_{a}^{2}=-1\right\}. \end{array}$$

Let *N* = ∑_*α*=*n*+2_^2*n*+2^ *x*_*i*_(∂/∂*x*_*i*_) be the normal vector field of *H*_*n*_^2*n*+1^. Then *G*(*N*, *N*) = −1.

Define a vector field *ξ*, a tensor field $\overset{-}{\phi }$ of type (1,1), a 1-form *η*, and a pseudo-Riemannian metric $\overset{-}{g}$ on *H*_*n*_^2*n*+1^ by assuming

(82) $$\begin{array}{c} J\left(X\right)=\overset{-}{\phi }X-\eta \left(X\right)N,\;\;\xi =-JN,\;\;\overset{-}{g}={G|}_{H_{n}^{2n+1}}. \end{array}$$

Then we get an almost paracontact metric structure $\left(\overset{-}{\phi },\xi ,\eta ,\overset{-}{g}\right)$ on *H*_*n*_^2*n*+1^. Moreover, this structure is para-Sasakian [11]. Now cut *H*_1_^3^ by the hyperplane *x*_1_ − *x*_3_ = 0 and obtain a lightlike surface *M* of *H*_1_^3^ with

(83) $$\begin{array}{c} \text{Rad}\;TM=\mathrm{Span}\left\{E=U_{1}\right\},\\ S\left(TM\right)=\mathrm{Span}\left\{W=-x_{1}U_{1}-U_{2}\right\}, \end{array}$$

where *U*_1_ = (∂/∂*x*_1_) + (∂/∂*x*_3_), *U*_2_ = *x*_4_(∂/∂*x*_2_) + *x*_2_(∂/∂*x*_4_) ∈ Γ(*TM*). It follows that the lightlike transversal bundle ltr(*TM*) is spanned by

(84) $$\begin{array}{c} N=\frac{1}{2}\left\{\frac{\partial }{\partial x_{1}}-\frac{2x_{1}}{x_{2}+x_{4}}\frac{\partial }{\partial x_{2}}-\frac{\partial }{\partial x_{3}}+\frac{2x_{1}}{x_{2}+x_{4}}\frac{\partial }{\partial x_{4}}\right\}. \end{array}$$

Furthermore,

(85) $$\begin{array}{c} \overset{-}{\phi }E=E,\;\;\overset{-}{\phi }N=-N. \end{array}$$

Thus *M* is an invariant surface of *H*_1_^3^.

## 5. Screen Semi-Invariant Lightlike Hypersurfaces of Almost Paracontact Metric Manifolds

If *E* is local section of Γ(Rad(*TM*)), one has $\overset{-}{g}(\overset{-}{\phi }E,E)=0$; therefore $\overset{-}{\phi }E\in \Gamma (TM)$ and we get a 1-dimensional distribution $\overset{-}{\phi }(\text{Rad}(TM))$ on *M*.

**Definition 18**

Let $\left(\overset{-}{M},\overset{-}{\phi },\xi ,\eta ,\overset{-}{g}\right)$ be a (2*n* + 1)-dimensional almost paracontact metric manifold and *M* a lightlike hypersurface of $\overset{-}{M}$. If

(86) $$\begin{array}{c} \overset{-}{\phi }\left(\text{Rad}\left(TM\right)\right)\subset S\left(TM\right),\\ \overset{-}{\phi }\left(\text{ltr}\left(TM\right)\right)\subset S\left(TM\right), \end{array}$$

then *M* will be called a screen semi-invariant lightlike hypersurface of $\overset{-}{M}$.

**Example 19**

Let $\overset{-}{M}=R^{7}$ be a 7-dimensional almost paracontact metric manifold with the structure $\left(\overset{-}{\phi },\xi ,\eta ,\overset{-}{g}\right)$ given in Example 1. Consider a hypersurface *M* of $\overset{-}{M}$ given by

(87) $$\begin{array}{c} x_{1}=y_{1}+x_{2}+y_{2}+x_{3}+y_{3}. \end{array}$$

Then the tangent bundle *TM* of *M* is spanned by

(88) {U1=∂∂x1+∂∂y1,U2=∂∂x1+∂∂x2,  U3=∂∂x1+∂∂y2,U4=∂∂x1+∂∂x3,  U5=∂∂x1+∂∂y3,U6=∂∂z}.

The radical distribution Rad *TM* and the lightlike transversal bundle ltr(*TM*) are given by

(89) Rad(TM)=Span⁡{E=U1−U2+U3−U4+U5},ltr(TM)=Span⁡{N=2∂∂x1+32∂∂y1+∂∂y2−12∂∂x3−∂∂y3}.

It follows that the screen distribution *S*(*TM*) is spanned by {*W*_1_, *W*_2_, *W*_3_, *W*_4_, *W*_5_}, where

(90) W1=U6,W2=U1+U2−U3+U4−U5,W3=U1−U3+U5,W4=−2U1−U2+U4+12U5,W5=4U1+U2−U4.

Furthermore,

(91) $$\begin{array}{c} \overset{-}{\phi }E=W_{2}\in \Gamma \left(S\left(TM\right)\right),\\ \overset{-}{\phi }N=-W_{4}\in \Gamma \left(S\left(TM\right)\right). \end{array}$$

Thus, *M* is a screen semi-invariant lightlike hypersurface of $\overset{-}{M}$.

From now on, we will write (*M*, *g*, *S*(*TM*)) to denote a screen semi-invariant lightlike hypersurface, together with the choices of a fixed nonzero section *E* of Rad*TM*, a fixed screen distribution *S*(*TM*), ltr(*TM*), and *N* as in Theorem 2.

Since *M* is a screen semi-invariant lightlike hypersurface, then we have $\overset{-}{\phi }N\in S(TM)$ and

(92) $$\begin{array}{c} \overset{-}{g}\left(\overset{-}{\phi }N,E\right)=-\overset{-}{g}\left(N,\overset{-}{\phi }E\right)=0,\;\;\overset{-}{g}\left(\overset{-}{\phi }N,N\right)=0, \end{array}$$

which imply that $\overset{-}{\phi }N$ is orthogonal to *S*(*TM*)^⊥^ by virtue of (19). Also, from (5), we obtain

(93) $$\begin{array}{c} \overset{-}{g}\left(\overset{-}{\phi }N,\overset{-}{\phi }E\right)=-1. \end{array}$$

Therefore, $\overset{-}{\phi }(\text{Rad}(TM))⊕\overset{-}{\phi }(\text{ltr}(TM))$ is a nondegenerate vector subbundle of *S*(*TM*) of rank 2.

In the following, being *S*(*TM*) and $\overset{-}{\phi }(\text{Rad}(TM))⊕\overset{-}{\phi }(\text{ltr}(TM))$ nondegenerate, we can define the unique nondegenerate distribution *D*_0_ such that [2]

(94) $$\begin{array}{c} S\left(TM\right)=\left\{\overset{-}{\phi }\left(\text{Rad}\left(TM\right)\right)⊕\overset{-}{\phi }\left(\text{ltr}\left(TM\right)\right)\right\}⊥D_{0}. \end{array}$$

Then *ξ* ∈ Γ(*D*_0_) and *D*_0_ is invariant under $\overset{-}{\phi }$; that is, $\overset{-}{\phi }(D_{0})=D_{0}$. Moreover, from (16), (20), and (94) we write

(95) $$\begin{array}{c} TM=\left\{\overset{-}{\phi }\left(\text{Rad}\left(TM\right)\right)⊕\overset{-}{\phi }\left(\text{ltr}\left(TM\right)\right)\right\}⊥D_{0}⊥\text{Rad}\left(TM\right), \end{array}$$

(96) TM−={ϕ−(Rad(TM))⊕ϕ−(ltr(TM))}⊥D0⊥{Rad(TM)⊕ltr(TM)}.

For an almost paracontact metric manifold $\left(\overset{-}{M},\overset{-}{\phi },\xi ,\eta ,\overset{-}{g}\right)$, we construct a useful local orthonormal basis. Let *U* be a coordinate neighborhood on $\overset{-}{M}$ and *e*_1_ any unit vector field on *U* orthogonal to *ξ*. Then $\overset{-}{\phi }e_{1}$ is a vector field orthogonal to both *e*_1_ and *ξ*, and $\overset{-}{g}(\overset{-}{\phi }e_{1},\overset{-}{\phi }e_{1})=-1$. Choose a unit vector field *e*_2_ orthogonal to *ξ*, *e*_1_, and $\overset{-}{\phi }e_{1}$. Then $\overset{-}{\phi }e_{2}$ is also orthogonal to *ξ*, *e*_1_, *e*_2_, and $\overset{-}{\phi }e_{1}$ and $\overset{-}{g}(\overset{-}{\phi }e_{2},\overset{-}{\phi }e_{2})=-1$. Proceeding in this way we obtain a set of local orthonormal vector fields $\left\{e_{i},\overset{-}{\phi }e_{i},\xi \right\}\;\;\left(i=1,2,\ldots ,n-2\right)$. Now construct the unit vector field $e_{n-1}=\left(E+N\right)/\sqrt{2}$ orthogonal to *ξ*, *e*_*i*_, and $\overset{-}{\phi }e_{i}$  (*i* = 1,2,…, *n* − 2). Then $\overset{-}{\phi }e_{n-1}=\left(\overset{-}{\phi }E+\overset{-}{\phi }N\right)/\sqrt{2}$ is also orthogonal to *ξ*, *e*_*i*_, and $\overset{-}{\phi }e_{i}$  (*i* = 1,2,…, *n* − 2) and $\overset{-}{g}(\overset{-}{\phi }e_{n-1},\overset{-}{\phi }e_{n-1})=-1$. By a similar way set a unit vector field $e_{n}=\left(\overset{-}{\phi }E-\overset{-}{\phi }N\right)/\sqrt{2}$ orthogonal to *ξ*, *e*_*i*_, and $\overset{-}{\phi }e_{i}\;\;\left(i=1,2,\ldots ,n-1\right)$. It is easy to see that $\overset{-}{\phi }e_{n}=\left(E-N\right)/\sqrt{2}$ is also orthogonal to *ξ*, *e*_*i*_, $\overset{-}{\phi }e_{i}\;\;\left(i=1,2,\ldots ,n-1\right)$ and $\overset{-}{g}(\overset{-}{\phi }e_{n},\overset{-}{\phi }e_{n})=-1$. Hence, from a quasi-orthonormal basis $\left\{e_{i},\overset{-}{\phi }e_{i},E,N,\overset{-}{\phi }E,\overset{-}{\phi }N,\xi \right\}\;\;\left(i=1,2,\ldots ,n-2\right)$ of $\overset{-}{M}$, we obtain a local orthonormal basis

(97) {ei,en−1=E+N2,en=ϕ−E−ϕ−N2, ϕ−ei,ϕ−en−1=ϕ−E+ϕ−N2,ϕ−en=E−N2,ξ},

where *i* = 1,2,…, *n* − 2, called $\overset{-}{\phi }$-basis. Thus we have the following.

**Proposition 20**

Let $\left(\overset{-}{M},\overset{-}{\phi },\xi ,\eta ,\overset{-}{g}\right)$ be a (2*n* + 1)-dimensional almost paracontact metric manifold and *M* a screen semi-invariant lightlike hypersurface of $\overset{-}{M}$. Then there always exists a $\overset{-}{\phi }$-basis on $\overset{-}{M}$ generated from a quasi-orthonormal basis $\left\{e_{i},\overset{-}{\phi }e_{i},E,N,\overset{-}{\phi }E,\overset{-}{\phi }N,\xi \right\},i=1,2,\ldots ,n-2$, of $\overset{-}{M}$.

Now, we consider the distributions $D=\text{Rad}(TM)⊥\overset{-}{\phi }(\text{Rad}(TM))⊥D_{0}$, $D^{\prime }=\overset{-}{\phi }(\text{ltr}(TM))$ on *M*. Then *D* is a $\overset{-}{\phi }$-invariant distribution and we have

(98) $$\begin{array}{c} TM=D⊕D^{\prime }. \end{array}$$

Thus, every *X* ∈ Γ(*TM*) can be expressed by

(99) $$\begin{array}{c} X=RX+QX, \end{array}$$

where *R* and *Q* are the projections of *TM* into *D* and *D*′, respectively. Hence, we may write $\phi X=\bar{\phi }RX$, for any *X* ∈ Γ(*TM*). Let us consider the local lightlike vector fields $U=\overset{-}{\phi }N\in \Gamma (\overset{-}{\phi }(\text{ltr}(TM)))\;$and $V=\overset{-}{\phi }E\in \Gamma D$. From (1), (38), and (39), we obtain

(100) $$\begin{array}{c} {\bar{\phi }}^{2}X=\phi^{2}X+u\left(X\right)U+u\left(\phi X\right)N. \end{array}$$

By comparing the tangential and transversal parts in (100) we get

(101) $$\begin{array}{c} \phi^{2}=I-\eta ⊗\xi -u⊗U, \end{array}$$

(102) $$\begin{array}{c} u\circ \phi =0, \end{array}$$

respectively. Next, from (3) one can easily see that

(103) $$\begin{array}{c} \phi \xi =0,\;\;u\left(\xi \right)=0. \end{array}$$

Since ${\bar{\phi }}^{2}N=N$, by using (38), we also have

(104) $$\begin{array}{c} \phi U=0,\;\;u\left(U\right)=1. \end{array}$$

Furthermore, from (4), we have

(105) $$\begin{array}{c} \eta \left(U\right)=0. \end{array}$$

Finally, we get

(106) $$\begin{array}{c} \left(\eta \circ \phi \right)X=\eta \left(\bar{\phi }X-u\left(X\right)N\right), \end{array}$$

which gives

(107) $$\begin{array}{c} \eta \circ \phi =0. \end{array}$$

Thus we have the following.

**Proposition 21**

Let *M* be a screen semi-invariant lightlike hypersurface of an almost paracontact metric manifold $\left(\overset{-}{M},\overset{-}{\phi },\xi ,\eta ,\overset{-}{g}\right)$. Then *M* possesses a para (*ϕ*, *ξ*, *η*, *U*, *u*)-structure; that is,

(108) $$\begin{array}{c} \phi^{2}=I-\eta ⊗\xi -u⊗U,\;\;\phi \xi =0,\\ \phi U=0,\;\;\eta \circ \phi =0,\\ u\circ \phi =0,\;\;\eta \left(\xi \right)=1,\;\;u\left(U\right)=1,\\ \eta \left(U\right)=0,\;\;u\left(\xi \right)=0. \end{array}$$

**Theorem 22**

Let *M* be a screen semi-invariant lightlike hypersurface of a para-Sasakian manifold $\left(\overset{-}{M},\overset{-}{\phi },\xi ,\eta ,\overset{-}{g}\right)$. Then one has

(109) $$\begin{array}{c} \left(\nabla_{X}\phi \right)Y=-g\left(X,Y\right)\xi +\eta \left(Y\right)X+u\left(Y\right)A_{N}X+B\left(X,Y\right)U, \end{array}$$

(110) $$\begin{array}{c} \left(\nabla_{X}u\right)Y=-B\left(X,\phi Y\right)-u\left(Y\right)\tau \left(X\right), \end{array}$$

for all *X*, *Y* ∈ Γ(*TM*).

**Proposition 23**

Let $\left(\overset{-}{M},\overset{-}{\phi },\xi ,\eta ,\overset{-}{g}\right)$ be a para-Sasakian manifold and (*M*, *g*, *S*(*TM*)) a screen semi-invariant lightlike hypersurface of $\overset{-}{M}$. Then M is totally geodesic if and only if, for any *X* ∈ Γ(*TM*) and for *Y* ∈ Γ(*D*),

(111) $$\begin{array}{c} \left(\nabla_{X}\phi \right)Y=-g\left(X,Y\right)\xi +\eta \left(Y\right)X, \end{array}$$

(112) $$\begin{array}{c} A_{N}X=-\phi \nabla_{X}u+g\left(X,U\right)\xi . \end{array}$$

**Proof**

Let assume that *M* is totally geodesic; that is, for any *X*, *Y* ∈ Γ(*TM*), *B*(*X*, *Y*) = 0. Then, for *Y* ∈ Γ(*D*) using *u*(*Y*) = 0 in (109) we have

(113) $$\begin{array}{c} \left(\nabla_{X}\phi \right)Y=-g\left(X,Y\right)\xi +\eta \left(Y\right)X. \end{array}$$

Similarly, using (109) we have

(114) $$\begin{array}{c} \left(\nabla_{X}\phi \right)U=A_{N}X-g\left(X,U\right)\xi +\eta \left(U\right)X, \end{array}$$

which gives

(115) $$\begin{array}{c} A_{N}X=-\phi \nabla_{X}U+g\left(X,U\right)\xi , \end{array}$$

by virtue of *ϕU* = 0 and *η*(*U*) = 0.

Conversely, suppose that the conditions (111) and (112) hold and we will prove that *B* vanishes. If *Y* ∈ Γ(*TM*), using the decomposition (98), there exists *α* ∈ *I*(*U*) such that

(116) $$\begin{array}{c} Y=Y_{D}+\alpha U \end{array}$$

and for any *X* ∈ Γ(*TM*) we obtain

(117) $$\begin{array}{c} B\left(X,Y\right)=B\left(X,Y_{D}\right)+\alpha B\left(X,U\right). \end{array}$$

For *Y* = *Y*_*D*_, using (109) and (111), we find

(118) $$\begin{array}{c} B\left(X,Y_{D}\right)=u\left(Y_{D}\right)A_{N}X=0, \end{array}$$

which implies that *B*(*X*, *Y*_*D*_) = 0.

Also, for any *Y* = *U* from (109), using (112), we get

(119) $$\begin{array}{c} B\left(X,U\right)U=0, \end{array}$$

which implies *B*(*X*, *U*) = 0. This completes the proof.

**Proposition 24**

Let $\left(\overset{-}{M},\overset{-}{\phi },\xi ,\eta ,\overset{-}{g}\right)$ be a para-Sasakian manifold and (*M*, *g*, *S*(*TM*)) a screen semi-invariant lightlike hypersurface of $\overset{-}{M}$. Then one has, for any *X* ∈ Γ(*TM*),

(i) *if the vector field U is parallel, then*
  (120) $$\begin{array}{c} A_{N}X=\eta \left(A_{N}X\right)\xi +u\left(A_{N}X\right)U,\;\tau \left(X\right)=0; \end{array}$$
(ii) *if the vector field V is parallel, then*
  (121) $$\begin{array}{c} A_{E}^{*}X=\eta \left(A_{E}^{*}X\right)\xi +u\left(A_{E}^{*}X\right)U,\;\tau \left(X\right)=0, \end{array}$$

where $U=\overset{-}{\phi }N$ and $V=\overset{-}{\phi }E$.

## 6. Integrability of Distributions on a Screen Semi-Invariant Lightlike Hypersurface of a Para-Sasakian Manifold

### 6.1. The Distribution *D*_0_

Firstly, we consider the distribution *D*_0_, defined in (94). Using (95) and putting $\mu =\{\overset{-}{\phi }(\text{Rad}(TM))⊕\overset{-}{\phi }(\text{ltr}(TM))\}⊥\text{Rad}(TM)$, for any *X* ∈ Γ(*TM*), *Y* ∈ Γ(*D*_0_), and *Z* ∈ Γ(*μ*) we have

(122) $$\begin{array}{c} \nabla_{X}Y={\overset{{}^{\circ}}{\nabla }}_{X}Y+\overset{{}^{\circ}}{h}\left(X,Y\right), \end{array}$$

(123) $$\begin{array}{c} \nabla_{X}Z=-{\overset{{}^{\circ}}{A}}_{Z}X+\nabla_{X}^{\mu }Z, \end{array}$$

where $\overset{{}^{\circ}}{\nabla }$ is a linear connection on the bundle *D*_0_, $\overset{{}^{\circ}}{h}:\Gamma (TM)\times \Gamma (D_{0})\to \Gamma (\mu )$ is an *I*(*M*) bilinear, $\overset{{}^{\circ}}{A}$ is an *I*(*M*) linear operator on Γ(*D*_0_), respectively, and ∇^*μ*^ is a linear connection on *μ*.

**Lemma 25**

Let (*M*, *g*, *S*(*TM*)) be a screen semi-invariant lightlike hypersurface of a para-Sasakian manifold $\left(\overset{-}{M},\overset{-}{\phi },\xi ,\eta ,\overset{-}{g}\right)$ and *U* ⊂ *M* a coordinate neighborhood as fixed in Theorem 2. Then, for any *X*, *Y* ∈ Γ(*D*_0_), one has

(124) $$\begin{array}{c} \overset{-}{g}\left(\left({\overset{-}{\nabla }}_{X}\overset{-}{\phi }\right)Y,E\right)=0, \end{array}$$

(125) $$\begin{array}{c} \overset{-}{g}\left(\left({\overset{-}{\nabla }}_{X}\overset{-}{\phi }\right)Y,N\right)=0. \end{array}$$

**Proof**

Calculation is straightforward by using (9).

Let *U* ⊂ *M* be a coordinate neighborhood as fixed in Theorem 2. Then according to decomposition given by (95) we set

(126) $$\begin{array}{c} \alpha_{1}\left(X,Y\right)=-g\left(\overset{{}^{\circ}}{h}\left(X,Y\right),\overset{-}{\phi }N\right),\\ \alpha_{2}\left(X,Y\right)=-g\left(\overset{{}^{\circ}}{h}\left(X,Y\right),\overset{-}{\phi }E\right),\\ \alpha_{3}\left(X,Y\right)=g\left(\overset{{}^{\circ}}{h}\left(X,Y\right),N\right), \end{array}$$

for any *X*, *Y* ∈ Γ(*D*_0_|_*U*_). So (122) can be written locally as

(127) $$\begin{array}{c} \nabla_{X}Y={\overset{{}^{\circ}}{\nabla }}_{X}Y+\alpha_{1}\left(X,Y\right)\overset{-}{\phi }E+\alpha_{2}\left(X,Y\right)\overset{-}{\phi }N+\alpha_{3}\left(X,Y\right)E. \end{array}$$

We will express *α*_1_, *α*_2_, and *α*_3_ in terms of *B* and *C*. Firstly, we compute

(128)   g(∇XY,ϕ−N)=g(∇°XY+α1(X,Y)ϕ−E+α2(X,Y)ϕ−N  +α3(X,Y)E,ϕ−N)=−α1(X,Y).

Then, by using (125), (24) and (25), we get

(129) g(∇XY,ϕ−N)=−g−(ϕ−∇XY,N)=−g−(ϕ−∇−XY,N)=g−((∇−Xϕ−)Y,N)−g−(∇−Xϕ−Y,N)=−g−(∇−Xϕ−Y,N)=g−(ϕ−Y,∇−XN)=−g−(ANX,ϕ−Y)=−C(X,ϕ−Y),

in view of *D*_0_ being $\overset{-}{\phi }$-invariant and $\overset{-}{\nabla }$ being a metric connection.

Next we have

(130) g(∇XY,ϕ−N)=g(∇°XY+α1(X,Y)ϕ−E+α2(X,Y)ϕ−N  +α3(X,Y)E,ϕ−E)=−α2(X,Y),

And, using (124), (24), and (31) in the previous equation, we obtain

(131) g(∇XY,ϕ−E)=−g−(ϕ−∇XY,E)=−g−(ϕ−∇−XY,E)=g−((∇−Xϕ−)Y,E)−g−(∇−Xϕ−Y,E)=−g−(∇−Xϕ−Y,E)=g−(ϕ−Y,∇−XE)=−B(X,ϕ−Y).

By a similar way, we compute

(132) g(∇XY,N)=g(∇°XY+α1(X,Y)ϕ−E+α2(X,Y)ϕ−N  +α3(X,Y)E,N)=α3(X,Y).

Since ∇_*X*_ *Y* = ∇_X_*Y + *C*(*X*, *Y*)*E*, then we get

(133) $$\begin{array}{c} g\left(\nabla_{X}Y,N\right)=C\left(X,Y\right). \end{array}$$

Therefore using the expressions of *α*_1_, *α*_2_, and *α*_3_ in (127) we write

(134) ∇XY=∇°XY+C(X,ϕ−Y)ϕ−E+B(X,ϕ−Y)ϕ−N+C(X,Y)E,

which implies that

(135) $$\begin{array}{c} \overset{{}^{\circ}}{h}\left(X,Y\right)=C\left(X,\overset{-}{\phi }Y\right)\overset{-}{\phi }E+B\left(X,\overset{-}{\phi }Y\right)\overset{-}{\phi }N+C\left(X,Y\right)E. \end{array}$$

**Theorem 26**

Let (*M*, *g*, *S*(*TM*)) be a screen semi-invariant lightlike hypersurface of a para-Sasakian manifold $\left(\overset{-}{M},\overset{-}{\phi },\xi ,\eta ,\overset{-}{g}\right)$. Then the distribution *D*_0_ is integrable if and only if

(136) $$\begin{array}{c} C\left(X,Y\right)=C\left(Y,X\right),\;\;B\left(X,\overset{-}{\phi }Y\right)=B\left(\overset{-}{\phi }X,Y\right),\\ C\left(X,\overset{-}{\phi }Y\right)=C\left(\overset{-}{\phi }X,Y\right), \end{array}$$

where *X*, *Y* ∈ Γ(*D*_0_).

**Proof**

Since ∇ is torsion free connection, by using (134), we have

(137) [X,Y]=∇°XY−∇°YX+(C(X,ϕ−Y)−C(ϕ−X,Y))ϕ−E+(B(X,ϕ−Y)−B(ϕ−X,Y))ϕ−N+(C(X,Y)−C(Y,X))E,

for any *X*, *Y* ∈ Γ(*D*_0_). Now suppose that *D*_0_ is integrable. Then the components of [*X*, *Y*] with respect to $\overset{-}{\phi }E$, $\overset{-}{\phi }N$, and *E* vanish. So, we get (136).

Conversely, if (136) is satisfied, then we get (137)

(138) $$\begin{array}{c} \left[X,Y\right]={\overset{{}^{\circ}}{\nabla }}_{X}Y-{\overset{{}^{\circ}}{\nabla }}_{Y}X, \end{array}$$

for any *X*, *Y* ∈ Γ(*D*_0_), which implies that [*X*, *Y*] ∈ Γ(*D*_0_). This completes the proof.

**Corollary 27**

Let $\left(\overset{-}{M},\overset{-}{\phi },\xi ,\eta ,\overset{-}{g}\right)$ be a para-Sasakian manifold and (*M*, *g*, *S*(*TM*)) a screen semi-invariant lightlike hypersurface of $\overset{-}{M}$. Then $\overset{{}^{\circ}}{h}$ is symmetric on *D*_0_ if and only if *D*_0_ is integrable.

**Theorem 28**

Let (*M*, *g*, *S*(*TM*)) be a screen semi-invariant lightlike hypersurface of a para-Sasakian manifold $\left(\overset{-}{M},\overset{-}{\phi },\xi ,\eta ,\overset{-}{g}\right)$ and the distribution *D*_0_ on *M* is integrable. Then *D*_0_ is minimal with respect to the symmetric connection ∇ on *M*; that is, $\text{trace}(\overset{{}^{\circ}}{h})=0$, if and only if

(139) $$\begin{array}{c} C\left(X,Y\right)=C\left(\overset{-}{\phi }X,\overset{-}{\phi }Y\right), \end{array}$$

for any *X*, *Y* ∈ Γ(*D*_0_).

**Proof**

Using decomposition (94), we find rank⁡*D*_0_ = 2*n* − 3. Now, from Proposition 20, consider an orthonormal $\overset{-}{\phi }$-basis $\left\{e_{i},\overset{-}{\phi }e_{i},\xi \right\}$ of *D*_0_, *i* = 1,2,…, *n* − 2. Then we have

(140) $$\begin{array}{c} \text{trace}\left(\overset{{}^{\circ}}{h}\right)=\overset{n-2}{\underset{i=1}{\sum }}\overset{{}^{\circ}}{h}\left(e_{i},e_{i}\right)-\overset{n-2}{\underset{i=1}{\sum }}\overset{{}^{\circ}}{h}\left(\overset{-}{\phi }e_{i},\overset{-}{\phi }e_{i}\right)+\overset{{}^{\circ}}{h}\left(\xi ,\xi \right). \end{array}$$

Also from (25) and (35) we have

(141) $$\begin{array}{c} \overset{{}^{\circ}}{h}\left(\xi ,\xi \right)=C\left(\xi ,\overset{-}{\phi }\xi \right)\overset{-}{\phi }E+B\left(\xi ,\overset{-}{\phi }\xi \right)\overset{-}{\phi }N+C\left(\xi ,\xi \right)E=0. \end{array}$$

Hence, using integrability condition of *D*_0_ we get

(142) $$\begin{array}{c} \overset{{}^{\circ}}{h}\left(e_{i},e_{i}\right)-\overset{{}^{\circ}}{h}\left(\overset{-}{\phi }e_{i},\overset{-}{\phi }e_{i}\right)=\left(C\left(e_{i},e_{i}\right)-C\left(\overset{-}{\phi }e_{i},\overset{-}{\phi }e_{i}\right)\right)E=0, \end{array}$$

which completes the proof.

Now, using the decomposition (96) and putting $\omega =\{\overset{-}{\phi }(\text{Rad}(TM))⊕\overset{-}{\phi }(\text{ltr}(TM))\}⊥\{\text{Rad}(TM)⊕\text{ltr}(TM)\}$, for any *X* ∈ Γ(*TM*), *Y* ∈ Γ(*D*_0_), and *Z* ∈ Γ(*ω*), we write

(143) $$\begin{array}{c} {\overset{-}{\nabla }}_{X}Y={\hat{\nabla }}_{X}Y+\hat{h}\left(X,Y\right), \end{array}$$

(144) $$\begin{array}{c} {\overset{-}{\nabla }}_{X}Z=-{\hat{A}}_{Z}X+\nabla_{X}^{\omega }Z, \end{array}$$

where $\hat{\nabla }$ is a linear connection on the bundle *D*_0_, $\hat{h}:\Gamma (TM)\times \Gamma (D_{0})\to \Gamma (\omega )$ is *I*(*M*) bilinear and $\hat{A}$ is an *I*(*M*) linear operator on Γ(*D*_0_), and ∇^*ω*^ is a linear connection on *ω*.

Let *U* ⊂ *M* be a coordinate neighborhood as fixed in Theorem 2. Then, by using (96), we set

(145) $$\begin{array}{c} \beta_{1}\left(X,Y\right)=-g\left(\hat{h}\left(X,Y\right),\overset{-}{\phi }N\right),\\ \beta_{2}\left(X,Y\right)=-g\left(\hat{h}\left(X,Y\right),\overset{-}{\phi }E\right),\\ \beta_{3}\left(X,Y\right)=g\left(\hat{h}\left(X,Y\right),N\right),\\ \beta_{4}\left(X,Y\right)=g\left(\hat{h}\left(X,Y\right),E\right), \end{array}$$

for any *X*, *Y* ∈ Γ(*D*_0_|_*U*_). Hence, (143) can be written locally:

(146) ∇−XY=∇^XY+β1(X,Y)ϕ−E+β2(X,Y)ϕ−N+β3(X,Y)E+β4(X,Y)N.

From the expression of *β*_*i*_, *i* = 1,2, 3,4, in terms of *B* and *C*, we obtain

(147) ∇−XY=∇^XY+C(X,ϕ−Y)ϕ−E+B(X,ϕ−Y)ϕ−N+C(X,Y)E+B(X,Y)N,

(148) h^(X,Y)=C(X,ϕ−Y)ϕ−E+B(X,ϕ−Y)ϕ−N+C(X,Y)E+B(X,Y)N.

**Theorem 29**

Let (*M*, *g*, *S*(*TM*)) be a screen semi-invariant lightlike hypersurface of a para-Sasakian manifold $\left(\overset{-}{M},\overset{-}{\phi },\xi ,\eta ,\overset{-}{g}\right)$ with the integrable distribution *D*_0_. Then *D*_0_ is minimal; that is, $\text{trace}(\hat{h})=0$, if and only if

(149) $$\begin{array}{c} C\left(X,Y\right)=C\left(\overset{-}{\phi }X,\overset{-}{\phi }Y\right),\;\;B\left(X,Y\right)=B\left(\overset{-}{\phi }X,\overset{-}{\phi }Y\right), \end{array}$$

for any *X*, *Y* ∈ Γ(*D*_0_).

**Proof**

Let $\left\{e_{i},\overset{-}{\phi }e_{i},\xi \right\}$, *i* = 1,2,…, *n* − 2, be an orthonormal $\overset{-}{\phi }$-basis. Then, we have

(150) $$\begin{array}{c} \text{trace}\left(\hat{h}\right)=\overset{n-2}{\underset{i=1}{\sum }}\hat{h}\left(e_{i},e_{i}\right)-\overset{n-2}{\underset{i=1}{\sum }}\hat{h}\left(\overset{-}{\phi }e_{i},\overset{-}{\phi }e_{i}\right)+\hat{h}\left(\xi ,\xi \right). \end{array}$$

It is easy to see that $\hat{h}(\xi ,\xi )=\overset{{}^{\circ}}{h}(\xi ,\xi )+B(\xi ,\xi )N=0$. Also, using (147), we get

(151)   h^(ei,ei)−h^(ϕ−ei,ϕ−ei)=(C(ei,ei)−C(ϕ−ei,ϕ−ei))E+(B(ei,ei)−B(ϕ−ei,ϕ−ei))N=0,

by virtue of integrability condition of *D*_0_. This completes the proof.

**Theorem 30**

Let $\left(\overset{-}{M},\overset{-}{\phi },\xi ,\eta ,\overset{-}{g}\right)$ be a para-Sasakian manifold and (*M*, *g*, *S*(*TM*)) a screen semi-invariant lightlike hypersurface of $\overset{-}{M}$. If *D*_0_ is integrable, then the leaves of *D*_0_ have a para-Sasakian structure.

**Proof**

Let (*M*, *g*, *S*(*TM*)) be a screen semi-invariant lightlike hypersurface and $\overset{{}^{\circ}}{M}$ a leaf of *D*_0_. Then, for any $p\in \overset{{}^{\circ}}{M}$, we have $T_{p}\overset{{}^{\circ}}{M}={\left(D_{0}\right)}_{p}$. Since *S* : Γ(*TM*) → Γ(*D*) and $D=D_{0}⊥\overset{-}{\phi }(\text{Rad}(TM))⊥\text{Rad}(TM)$, we get

(152) $$\begin{array}{c} \phi X_{0}=\overset{-}{\phi }SX_{0}=\overset{-}{\phi }X_{0}, \end{array}$$

for any $X_{0}\in T\overset{{}^{\circ}}{M}$.

By putting $\overset{{}^{\circ}}{\phi }={\phi |}_{D_{0}}$ and $\overset{{}^{\circ}}{\eta }={\eta |}_{D_{0}}$, one can see that $\overset{{}^{\circ}}{\phi }$ defines an (1,1)-tensor field on $\overset{{}^{\circ}}{M}$ because *D*_0_ is $\overset{-}{\phi }$-invariant. From (101), we get

(153) $$\begin{array}{c} \overset{{}^{\circ}}{\phi }X_{0}=X_{0}-\overset{{}^{\circ}}{\eta }\left(X_{0}\right)\xi ,\\ \overset{{}^{\circ}}{\eta }\left(\xi \right)=1, \end{array}$$

for any $X_{0}\in T\overset{{}^{\circ}}{M}$. Hence $\left(\overset{{}^{\circ}}{M},\overset{{}^{\circ}}{\phi },\xi ,\overset{{}^{\circ}}{\eta }\right)$ is an almost paracontact manifold.

Next, by using (38), for any $X_{0},Y_{0}\in \Gamma (T\overset{{}^{\circ}}{M})$, we have

(154) g(ϕ°X0,ϕ°Y0)=g−(ϕ−X0−u(X0)N,ϕ−Y0−u(Y0)N)=−g(X0,Y0)+η°(X0)η°(Y0),

which implies that $\left(\overset{{}^{\circ}}{M},\overset{{}^{\circ}}{\phi },\xi ,\overset{{}^{\circ}}{\eta },g\right)$ is an almost paracontact metric manifold.

Moreover, for any $X_{0},Y_{0}\in \Gamma (T\overset{{}^{\circ}}{M})$, $d\eta (X_{0},Y_{0})=d\overset{{}^{\circ}}{\eta }(X_{0},Y_{0})$, and $\overset{{}^{\circ}}{\Phi }(X_{0},Y_{0})=g(X_{0},\overset{{}^{\circ}}{\phi }Y_{0})=$ $\overset{-}{g}(X_{0},\overset{{}^{\circ}}{\phi }Y_{0})=d\eta (X_{0},Y_{0})=d\overset{{}^{\circ}}{\eta }(X_{0},Y_{0})$. Also $N_{\overset{{}^{\circ}}{\phi }}+2d\overset{{}^{\circ}}{\eta }⊗\xi$ and $N\overset{-}{\phi }+2d\overset{-}{\eta }⊗\xi$ coincide on *D*_0_.

Finally, we have

(155) (∇°X0g)(Y0,Z0)=X0g(Y0,Z0)−g(∇°X0Y0,Z0)−g(Y0,∇°X0Z0)=X0g(Y0,Z0)−g(∇X0Y0,Z0)+g(h°(X0,Y0),Z0)−g(Y0,∇X0Z0)+g(Y0,h°(X0,Z0))=(∇X0g)(Y0,Z0)=0,

for any $X_{0},Y_{0},Z_{0}\in \Gamma (T\overset{{}^{\circ}}{M})$, which implies that $\overset{{}^{\circ}}{\nabla }$ is a Levi-Civita connection and using (5) we get

(156) $$\begin{array}{c} \left({\overset{{}^{\circ}}{\nabla }}_{X_{0}}\overset{{}^{\circ}}{\phi }\right)Y_{0}=-g\left(X_{0},Y_{0}\right)\xi +\eta \left(Y_{0}\right)X_{0}. \end{array}$$

This completes the proof.

### 6.2. Integrability of *D*

In this section, we consider the distribution *D*, which is defined by $D=\text{Rad}(TM)⊥\overset{-}{\phi }(\text{Rad}(TM))⊥D_{0}$. Firstly we have the following.

**Lemma 31**

Let $\left(\overset{-}{M},\overset{-}{\phi },\xi ,\eta ,\overset{-}{g}\right)$ be a para-Sasakian manifold and  (*M*, *g*, *S*(*TM*))  a screen semi-invariant lightlike hypersurface of $\overset{-}{M}$. Then, for any *X* ∈ Γ(*TM*) and $Y\in \Gamma (T\overset{-}{M})$, one has

(157) $$\begin{array}{c} \overset{-}{g}\left(\left({\overset{-}{\nabla }}_{X}\overset{-}{\phi }\right)Y,E\right)=0; \end{array}$$

that is, the component of $({\overset{-}{\nabla }}_{X}\overset{-}{\phi })Y$ along ltr(*TM*) vanishes.

**Proposition 32**

Let $\left(\overset{-}{M},\overset{-}{\phi },\xi ,\eta ,\overset{-}{g}\right)$ be a para-Sasakian manifold and  (*M*, *g*, *S*(*TM*))  a screen semi-invariant lightlike hypersurface of $\overset{-}{M}$. Then the distribution *D* is integrable if and only if *B* satisfies the following conditions:

- $B(X,\overset{-}{\phi }Y)=B(\overset{-}{\phi }X,Y)$,* for any X*, *Y* ∈ Γ(*D*_0_),
- *B*(*X*, *V*) = 0,* for any X* ∈ Γ(*D*_0_),
- *B*(*V*, *V*) = 0,

where $V=\overset{-}{\phi }E$.

**Proof**

For any *X*, *Y* ∈ Γ(*D*), we obtain the component of [*X*, *Y*] along $\overset{-}{\phi }\text{ltr}(TM)$ as

(158) $$\begin{array}{c} \overset{-}{g}\left(\left[X,Y\right],\overset{-}{\phi }E\right)=g\left(\overset{-}{\phi }X,A_{E}^{*}Y\right)-g\left(\overset{-}{\phi }Y,A_{E}^{*}X\right). \end{array}$$

From the definition of the distribution *D*, we set

(159) $$\begin{array}{c} X=X_{0}+\sigma_{1}E+\sigma_{2}\overset{-}{\phi }E,\;\;Y=Y_{0}+\rho_{1}E+\rho_{2}\overset{-}{\phi }E. \end{array}$$

Since *D* is $\overset{-}{\phi }$-invariant, from the previous expressions of *X*, *Y* ∈ Γ(*D*) and from (26), we have

(160)   g−([X,Y],ϕ−E)=(σ1ρ2−σ2ρ1)B(V,V)−ρ2B(ϕ−X0,V)+σ2B(ϕ−Y0,V)−σ1B(Y0,V)+ρ1B(X0,V)−B(X0,ϕ−Y0)+B(Y0,ϕ−X0),

for any *X*, *Y* ∈ Γ(*TM*).

Now assume that *D* is integrable. Since $\overset{-}{\phi }E$, *E*, *X*_0_, and *Y*_0_ are sections of *D*, then we get

(161) $$\begin{array}{c} 0=\overset{-}{g}\left(\left[\overset{-}{\phi }E,E\right],\overset{-}{\phi }E\right)=-\overset{-}{g}\left(\overset{-}{\phi }E,\overset{-}{\phi }E\right)=-B\left(V,V\right). \end{array}$$

If *X* ∈ Γ(*D*_0_), we find

(162) $$\begin{array}{c} 0=\overset{-}{g}\left(\left[X,E\right],\overset{-}{\phi }E\right)=B\left(E,\overset{-}{\phi }X\right)-B\left(X,\overset{-}{\phi }E\right)=-B\left(X,V\right), \end{array}$$

and if *X*, *Y* ∈ Γ(*D*_0_) we get

(163) $$\begin{array}{c} 0=\overset{-}{g}\left(\left[X,Y\right],\overset{-}{\phi }E\right)=B\left(\overset{-}{\phi }Y,X\right)-B\left(\overset{-}{\phi }X,Y\right). \end{array}$$

Consequently using (160) with (i), (ii), and (iii), it is easy to check that [*X*, *Y*] in Γ(*D*). This completes the proof.

**Proposition 33**

Let $\left(\overset{-}{M},\overset{-}{\phi },\xi ,\eta ,\overset{-}{g}\right)$ be a para-Sasakian manifold and  (*M*, *g*, *S*(*TM*))  be a screen semi-invariant lightlike hypersurface of $\overset{-}{M}$. If (*M*, *g*, *S*(*TM*)) is totally geodesic, then the following statements hold.

- *The distribution D is integrable.*
- *The distribution D is parallel with respect to the induced connection *∇.

**Proof**

(i) Assume that (*M*, *g*, *S*(*TM*)) is totally geodesic. Then we can state that the distribution *D* is integrable from Proposition 32.

(ii) For any *X* ∈ Γ(*TM*) and *Y* ∈ Γ(*D*_0_), using (157), we get

(164) g(∇XE,ϕ−E)=g−(∇−XE,ϕ−E)=−g−(E,∇−Xϕ−E)=B(X,ϕ−E)=0,g(∇Xϕ−E,ϕ−E)=g−(∇−Xϕ−E,ϕ−E)=−g−(ϕ−∇−Xϕ−E,E)=g−((∇−Xϕ−)ϕ−E,E)−g−(∇−Xϕ−2E,E)=g(∇XE,E)=0,g(∇XY0,ϕ−E)=g−(∇−XY0,ϕ−E)=−g−(ϕ−∇−XY0,E)=g−((∇−Xϕ−)Y0,E)−g−(∇−Xϕ−Y0,E)=−g−(∇−Xϕ−Y0,E)=−B(X,ϕ−Y0)=0.

This completes the proof.

## References

[1] Duggal KL, Bejancu A (1996). *Lightlike Submanifolds of Semi-Riemannian Manifolds and Applications*.

[2] Duggal KL, Şahin B (2010). *Differential Geometry of Lightlike Submanifolds*.

[3] Kaneyuki S, Konzai M (1985). Paracomplex structure and affine symmetric spaces. *Tokyo Journal of Mathematics*.

[4] Zamkovoy S (2009). Canonical connections on paracontact manifolds. *Annals of Global Analysis and Geometry*.

[5] Alekseevsky DV, Cortés V, Galaev AS, Leistner T (2009). Cones over pseudo-Riemannian manifolds and their holonomy. *Journal fur die Reine und Angewandte Mathematik*.

[6] Alekseevsky DV, Medori C, Tomassini A (2006). Maximally homogeneous para-CR manifolds. *Annals of Global Analysis and Geometry*.

[7] Cortés V, Mayer C, Mohaupt T, Saueressing F (2004). Special geometry of Euclidean supersymmetry 1. Vector multiplets. *Journal of High Energy Physics*.

[8] Cortés V, Lawn M-A, Schäfer L (2006). Affine hyperspheres associated to special para-Kähler manifolds. *International Journal of Geometric Methods in Modern Physics*.

[9] Erdem S (2002). On almost (para)contact (hyperbolic) metric manifolds and harmonicity of (*φ*, *φ*′)-holomorphic maps between them. *Houston Journal of Mathematics*.

[10] O'Neill B (1983). *Semi-Riemannian Geometry with Applications to Relativity*.

[11] Wełyczko J Para CR-Structure on almost paracontact metric manifolds. http://arxiv.org/abs/1202.6383.

